# The Efficacy and Safety of Ginkgo Terpene Lactone Preparations in the Treatment of Ischemic Stroke: A Systematic Review and Meta-Analysis of Randomized Clinical Trials

**DOI:** 10.3389/fphar.2022.821937

**Published:** 2022-03-18

**Authors:** Huan Zhao, Qiang Guo, Baoli Li, Min Shi

**Affiliations:** ^1^ Department of Neurology, Hospital of Chengdu University of Traditional Chinese Medicine, Chengdu, China; ^2^ Department of Geriatrics, Chengdu First People’s Hospital, Chengdu, China; ^3^ Department of Neurology, Chengdu University of Traditional Chinese Medicine, Chengdu, China

**Keywords:** ischemic stroke, ginkgo terpene lactone injections, meta-analysis, efficacy, safety

## Abstract

**Background:** This meta-analysis aimed to assess the efficacy and safety of ginkgo terpene lactone preparations including ginkgo diterpene lactone meglumine injection, ginkgolide injection, and ginkgolide B injection for ischemic stroke (IS).

**Methods:** We searched the randomized controlled trials (RCTs) with publication date earlier than 31 August 2021 in PubMed, China National Knowledge Infrastructure (CNKI), Chinese Science and Technology Journals Database (VIP), Chinese Biomedical Literature Database (CBM), Wanfang Database, Embase, and the Cochrane Library. RevMan 5.3 software was applied to analyze the data and generate the forest plot and funnel plot. Meanwhile, publication bias was also assessed by Egger’s test with STATA 12 software.

**Results:** A total of 28 RCTs were eligible for inclusion. Among them, 23 RCTs were used to evaluate the efficacy of ginkgo terpene lactone preparations as the main treatment intervention for IS. To be specific, ginkgo diterpene lactone meglumine injection was superior to control drug in improving clinical efficacy [RR = 1.18, 95% CI (1.12, 1.24), Z = 6.36, *p* < 0.001] and neurological function [MD = −1.42, 95% CI (−1.91, −0.93), Z = 5.66, *p* < 0.001]. However, the effectiveness of the ginkgolide B injection group was equivalent to that of the control group. Additionally, ginkgolide injection achieved better clinical efficacy [RR = 1.10, 95% CI (1.02, 1.18), Z = 2.36, *p* = 0.02], but the changes of neurological function deficit was not obviously different between two groups [MD = −0.43, 95% CI (−4.32, 3.46), Z = 0.22, *p* = 0.83]. Furthermore, meta-analysis of five trials on ginkgo diterpene lactone meglumine injection combined with recombinant tissue plasminogen activator (rt-PA) thrombolytic therapy for acute IS showed that combination therapy was better in improving clinical efficacy [OR = 1.91, 95% CI (1.13, 3.22), Z = 2.41, *p* = 0.02] and neurological function [MD = −3.31, 95% Cl (−3.64,−2.98), Z = 19.63, *p* < 0.001]. Importantly, no serious adverse drug reactions/adverse drug events (ADRs/ADEs) were reported.

**Conclusion:** Ginkgo terpene lactone preparations have good therapeutic effects on patients with IS. For acute IS, ginkgo diterpene lactone meglumine injection can be used as a complementary therapy to improve the clinical efficacy of rt-PA.

## Introduction

Ischemic stroke (IS) is the most common type of cerebrovascular disease, which refers to ischemic necrosis and softening of brain tissue due to brain blood circulation impairment, ischemia, and hypoxia. The clinical symptoms present disturbance of consciousness, hemiplegia, aphasia, dysphagia, and blindness (Cerebrovascular Disease Group, 2010). Currently, the main approaches to treat IS include platelet aggregation therapy, thrombolytic therapy, nutritive therapy, and reducing intracranial pressure, blood pressure, and blood sugar and lipids (Barthels and Das, 2020). However, the efficacy and prognosis were not satisfied. Thus, exploring new treatments to improve poststroke recovery is urgently needed.

Traditional Chinese medicine therapy is widely used in treatment for multiple diseases including stroke (Luo et al., 2019). Ginkgo terpene lactones are a class of compounds extracted from *Ginkgo biloba* leaf, mainly including ginkgolides and bilobalide. Ginkgolides belong to diterpene lactones, which mainly include ginkgolides A, B, C, K, J, L, M, N, P, and Q (Zeng et al., 2013; Du, 2018). Ginkgolides A, B, C, and K, the main active compounds, are also platelet-activating factor (PAF) receptor antagonists (Lachachi et al., 1985). Additionally, PAF exerts the vital roles on cardiovascular pathophysiological processes, which have been proved (Braquet and Hosford, 1991; Montrucchio et al., 2000). Furthermore, previous studies have confirmed that its related preparations (ginkgolide injection, ginkgo diterpene lactone meglumine injection, and ginkgolide B injection, etc.) have good efficacy in treating cardiovascular and cerebrovascular diseases (Nabavi et al., 2015; Luo et al., 2017; Dong et al., 2020). To be specific, ginkgolide injection contains bilobalide, ginkgolide A, ginkgolide B, and ginkgolide C. Ginkgo diterpene lactone meglumine injection is composed of ginkgolide A, ginkgolide B, and ginkgolide K. Additionally, ginkgolide B injection is the only ginkgolide monomer injection in the world (purity>99%). The aforementioned three injections are widely used for IS caused by blood stasis blocking meridians in clinical practice. In general, ginkgo diterpene lactone meglumine injection is superior to ginkgolide injection and ginkgolide B injection because it is a reasonable combination of ginkgolide A, ginkgolide B, and ginkgolide K monomers with the strongest diterpene activity (Xu et al., 2017).

Although ginkgo terpene lactone injections have been shown to have good efficacy on IS *via* several randomized controlled trials (RCTs), the main effects and corresponding add-on effects of these injections have not been systematically evaluated. The current study aimed to conduct a meta-analysis of RCTs of ginkgo terpene lactone injections in the treatment of IS and then systematically evaluate their effects and safeties. Those findings may provide guidance for clinical decision-making.

## Materials and Methods

### Searching Strategy

A systematic search was conducted in several databases including PubMed, China National Knowledge Infrastructure (CNKI), Chinese Science and Technology Journals Database (VIP), Chinese Biomedical Literature Database (CBM), Wanfang Database, Embase, and the Cochrane Library. The search time ranged from the establishment time of each database to 31 August 2021. The screening language was either Chinese or English. The method of combining subject words and free words was used for searching articles such as (“Ischemic stroke” OR “Cerebral infarction”) AND (“Ginkgo terpene lactone injection” OR “Ginkgolides” OR “Ginkgolide A, BN52020” OR “Ginkgolide B, BN52021” OR “Ginkgolide C, BN52022” OR “Ginkgolide M, BN52023” OR “Ginkgolide J, BN52024).

### Inclusion Criteria

Inclusion criteria were abided by the participants, interventions, comparison/control, outcomes, and study design (PICOS) format, which were as follows: 1) Participants in the study were adults (age ≥18 years) with IS according to diagnostic criteria, regardless of gender or ethnicity; 2) Study design: RCTs in Chinese or English; 3) Intervention measures: The experimental groups were used for ginkgo terpene lactone preparations or recombinant tissue plasminogen activator (rt-PA) thrombolytic therapy on the basis of ginkgo terpene lactone preparations; 4) The control group received only other types of drugs, and the form of drugs was not limited; 5) The outcome measures met the following primary or secondary outcomes: the primary outcomes were the clinical efficacy defined according to the nationally approved criteria (The Fourth National Conference on cerebrovascular diseases, 1996). To be specific, basic heal was indicated when the decreases of neurological function deficit were between 90 and 100%, and the level of sick was “0.” The decreases of neurological function deficit were between 46 and 90%, and the level of sick was between “1” and “3,” indicating significant progresses. Progress was determined when the decreases of neurological function deficit were between 18 and 45%. Ineffectiveness was determined when the decreases of neurological function deficit were <17%. The secondary outcomes were the changes of neurological function deficit evaluated by the National Institute of Health Stroke Scale (NIHSS) (Brott et al., 1989) or Chinese clinical neurological function deficit scale (NFDS) (The Fourth National Conference on cerebrovascular diseases, 1996) and adverse drug reactions/adverse drug events (ADRs/ADEs).

### Exclusion Criteria

The exclusion criteria were 1) animal experiments, 2) repeated publications, 3) review, 4) no clear inclusion and exclusion criteria or incomplete inclusion and exclusion criteria, and 5) other diseases.

### Data Extraction and Quality Assessment

In total, two independent reviewers conducted the literature screening and data extraction. The following data were retrieved: title, author, publication date, sample size, baseline characteristics, interventions, course of treatment, outcome evaluation indicators, and ADRs/ADEs. If any disagreement arose, it was resolved through discussion with the third evaluator.

Quality assessment was performed by the Cochrane risk-of-bias tool, which consisted of random allocation method, allocation concealment, blinding methods, incomplete outcomes, and other biases. Each study was rated “yes,” “no,” and “unclear”. “Yes” indicated the low risk of bias. “No” indicated the high risk of bias. “Unclear” indicated the unclear risk of bias.

### Statistical Analysis

For this meta-analysis, data were analyzed by RevMan 5.3 software or STATA 12.0 software. RevMan 5.3 software was utilized to analyze the outcomes including clinical efficacy, the changes of neurological function deficit, and ADRs/ADEs. Heterogeneity was evaluated using the chi-square (chi^2^) test and I-square (I^2^) test. When the homogeneity was high (*p* > 0.10, I^2^<50%), the fixed effect model was used for analysis. When *p* < 0.10 and I^2^ ≥ 50%, the random effect model was used for analysis. Measurement data were presented as mean difference (MD), and categorical data were presented as odds ratio (OR) or relative risk (RR) with 95% confidence interval (95% CI). Additionally, publication bias was evaluated by funnel plots with RevMan 5.3 software and Egger’s test with STATA 12.0 software.

## Results

### Study Selection Process

**FIGURE 1:**
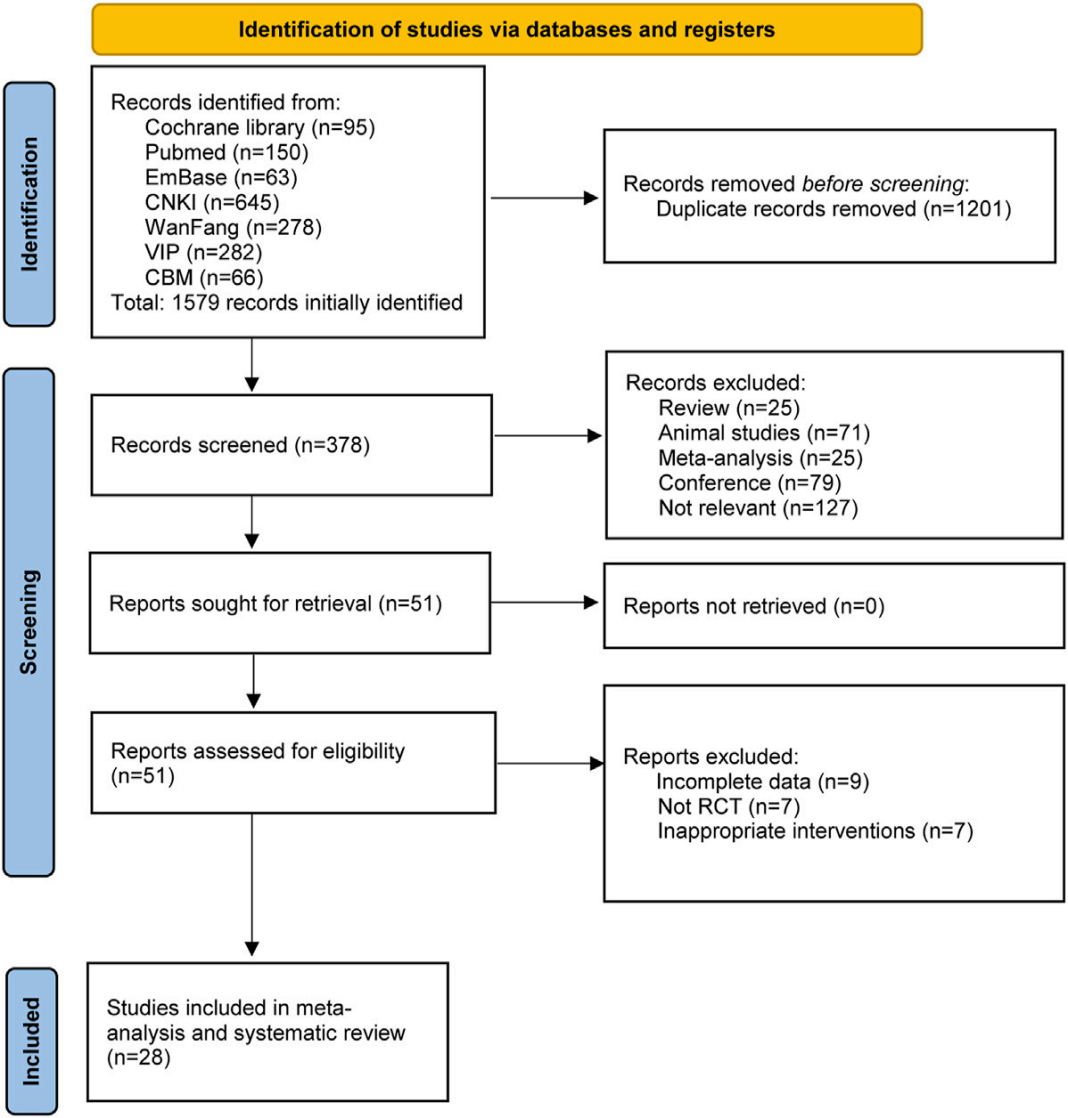
Flowchart of literature search and selection process.

According to the search strategy, a total of 1,579 references were initially retrieved. After the duplicate articles were removed, the remaining 378 articles were filtered according to the inclusion and exclusion criteria, and then 327 articles were excluded. With further reading the full text, 28 studies were finally included for meta-analysis. The screening process is shown in Figure 1.

### Characteristics of Included Studies

The basic characteristics of ginkgo terpene lactone preparations used in 28 studies are listed in Table 1. Among them, 23 RCTs were used to evaluate the effects of ginkgo terpene lactone preparations as the main treatment intervention for IS. Specifically, 23 studies involving 2,389 patients were as follows: Ren and Zhao (2006); Gu (2008); Liu (2008); Zheng (2009); Zhao et al. (2013); Xia (2014); Gao et al. (2015); Hua (2015); Ji and Cheng (2015); Qiu and Xiao (2015); Chen (2016); Zhang (2016); Zhang et al. (2017); Guan (2018); Jiang et al. (2018); Zhang et al. (2018); Zhang and Peng (2018); Zhu et al. (2019); Feng et al. (2020); Lu (2020); Lu et al. (2020); Zhou et al. (2020); Zhu et al. (2020). Additionally, five references (Kong et al., 2020; Zhai et al., 2020; Zhang et al., 2020; Zhou, 2020; Yu et al., 2021) involving 533 patients were included, all of whom were injected with ginkgo diterpene lactone meglumine and rt-PA to evaluate the add-on effect of ginkgo terpene lactone injection on acute IS.

**TABLE 1:** Basic characteristics of included studies.

Study	Case (*n*)		Average age (years)		Gender (Male/female)		Intervening measure		Effectiveness (*n*)		Course of treatment (days)	Outcome	Publication country	Publication language
	E	C	E	C	E	C	E	C	E	C	-	-	-	-
Guan (2018)	40	40	56.9	44.6	32/8	30/10	Ginkgo diterpene lactone meglumine injection	Ginkgo dipyridolum injection	38	31	14	1, 3	China	Chinese
Lu et al. (2020)	36	36	52.2	51.8	20/16	19/17	Ginkgo diterpene lactone meglumine injection	Aspirin + atorvastatin	-	-	14	2, 3	China	Chinese
Zhu et al. (2019)	41	41	65.9	63.5	28/13	29/12	Ginkgo diterpene lactone meglumine injection	Xueshuantong injection	40	30	14	1	China	Chinese
Zhang et al. (2018)	53	50	62.1	61.5	38/15	35/15	Ginkgo diterpene lactone meglumine injection	Danhong injection	-	-	14	2, 3	China	Chinese
Jiang et al. (2018)	50	50	53.9	53.8	27/23	28/22	Ginkgo diterpene lactone meglumine injection	Xueshuantong injection	48	41	14	1, 3	China	Chinese
Zhang et al. (2018)	43	43	64.4	66.2	26/17	27/16	Ginkgo diterpene lactone meglumine injection	Shuxuening injection	38	28	14	1, 2, 3	China	Chinese
Zhu et al. (2020)	61	66	67.2	66.6	42/19	42/24	Ginkgo diterpene lactone meglumine injection	Dengzhanxixin injection	57	54	14	1, 2, 3	China	Chinese
Zhou (2020)	106	56	64.3	62.1	60/46	39/17	Ginkgolide injection	Butylphthalide injection	94	49	14	1, 3	China	Chinese
Feng et al. (2020)	53	53	49.6	50.1	-	-	Ginkgo diterpene lactone meglumine injection	Butylphthalide injection	48	45	14	1, 2	China	Chinese
Ren and Zhao (2006)	24	24	-	-	15/9	13/11	Ginkgolide injection	Shuxuening injection	23	21	14	1	China	Chinese
Zheng (2009)	30	30	-	-	16/14	17/13	Ginkgolide B injection	Ginaton injection	26	25	14	1, 2	China	Chinese
Gu (2008)	72	24	-	-	-	-	Ginkgolide injection	Shuxuening injection	63	19	14	1, 3	China	Chinese
Liu (2008)	24	24	56.0	54.7	16/8	14/10	Ginkgolide injection	Shuxuening injection	22	20	14	1, 2, 3	China	Chinese
Zhao et al. (2013)	308	101	-	-	-	-	Ginkgo diterpene lactone meglumine injection	Shuxuening injection	263	74	14	1, 2, 3	China	Chinese
Xia (2014)	24	30	58.7	62.6	18/6	18/12	Ginkgolide injection	Ginkgo leaf extract and dipyridamole injection	7	10	14	1, 2, 3	China	Chinese
Ji and Cheng (2015)	32	28	-	-	-	-	Ginkgolide B injection	Compound Danshen injection	28	20	14	1, 2	China	Chinese
Qiu and Xiao (2015)	32	31	57.4	56.3	19/13	17/14	Ginkgo diterpene lactone meglumine injection	Shuxuening injection	26	22	14	1, 2, 3	China	Chinese
Gao et al. (2015)	40	40	55.4	56.2	30/10	32/8	Ginkgo diterpene lactone meglumine injection	Ginkgo leaf extract and dipyridamole injection	38	31	14	1, 2, 3	China	Chinese
Hua (2015)	120	40	64.3	62.6	78/42	24/16	Ginkgo diterpene lactone meglumine injection	Shuxuening injection	102	29	14	1, 2, 3	China	Chinese
Chen (2016)	30	30	-	-	-	-	Ginkgo diterpene lactone meglumine injection	Ligustrazine injection	28	25	14	1	China	Chinese
Zhang (2016)	66	66	58.5	58.4	36/30	37/29	Ginkgolide injection	Ligustrazine injection	64	52	14	1, 3	China	Chinese
Zhang et al. (2017)	51	50	68.2	39.8	24/37	28/22	Ginkgo diterpene lactone meglumine injection	Shuxuening injection	-	-	14	2	China	Chinese
Lu (2020)	50	50	56.2	56.2	28/22	27/23	Ginkgo diterpene lactone meglumine injection	Xueshuantong injection	49	44	14	1	China	Chinese
Zhou (2020)	48	48	64.1	64.3	26/22	25/23	Ginkgo diterpene lactone meglumine + rt-PA	rt-PA	-	-	14	2, 3	China	Chinese
Kong et al. (2020)	54	54	65.2	64.9	30/24	33/21	Ginkgo diterpene lactone meglumine + rt-PA	rt-PA	51	43	14	1, 3	China	Chinese
Zhai et al. (2020)	78	64	66.2	65.8	42/36	38/26	Ginkgo diterpene lactone meglumine + rt-PA	rt-PA	54	31	14	1, 3	China	Chinese
Zhang et al. (2020)	45	45	55.2	55.2	24/21	23/22	Ginkgo diterpene lactone meglumine + rt-PA	rt-PA	-	-	14	2	China	Chinese
Yu et al. (2021)	51	46	65.1	64.7	27/24	24/22	Ginkgo diterpene lactone meglumine + rt-PA	rt-PA	45	34	14	1, 2, 3	China	Chinese

Note: E, experimental group; C, control group; 1, clinical efficacy; 2, changes of neurological function deficit; 3, ADRs/ADEs, adverse drug reactions/adverse drug events; -, not mentioned.

Across all studies, the average age of the patients was approximately between 40 and 70 years. There were more male participants than female participants. In addition, the characteristics such as course of treatment in different groups were similar.

### Quality Evaluation on Included Studies

**FIGURE 2:**
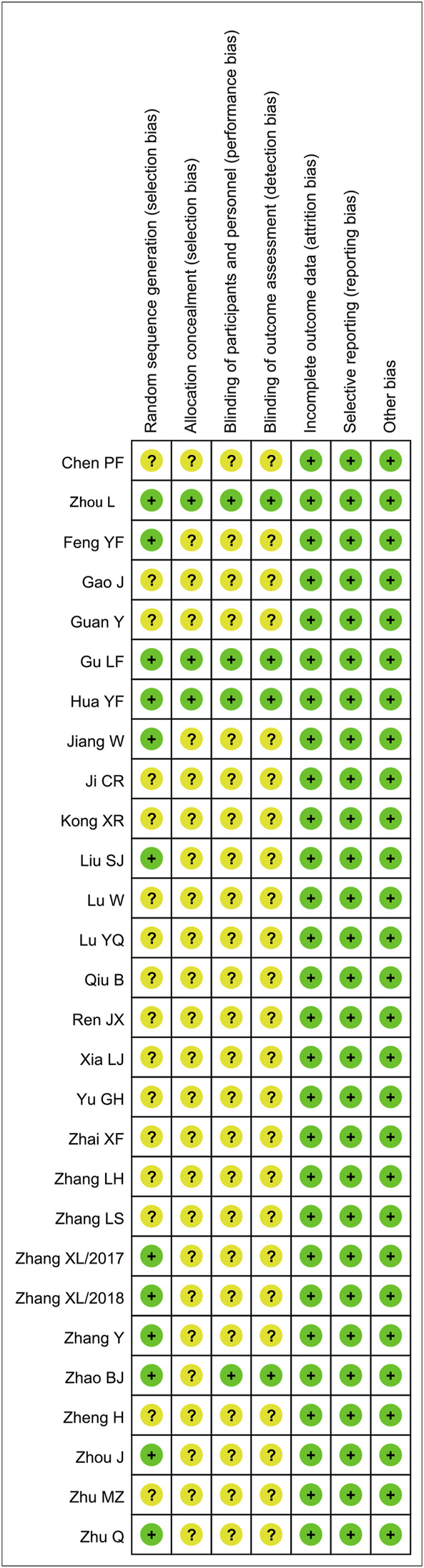
Quality evaluation of included literatures.

The quality of each of the 28 RCTs is shown in Figure 2. Although all articles mentioned “random,” only 12 studies described the specific randomization method (Gu, 2008; Liu, 2008; Zhao et al., 2013; Hua, 2015; Zhang, 2016; Jiang et al., 2018; Zhang et al., 2018; Feng et al., 2020; Zhang et al., 2020; Zhou, 2020; Zhou et al., 2020; Zhu et al., 2020). A total of three trials described the random allocation plan (Gu, 2008; Hua, 2015; Zhou et al., 2020), and the remaining trials did not report it. There were only four trials (Gu, 2008; Zhao et al., 2013; Hua, 2015; Zhou et al., 2020) using the blind method to evaluate the research objects and outcomes. All articles had the complete outcome data and did not describe the other risk biases.

### Meta-Analysis of Ginkgo Terpene Lactone Preparations as the Main Treatment Intervention for IS

#### Analysis of Clinical Efficacy

**FIGURE 3:**
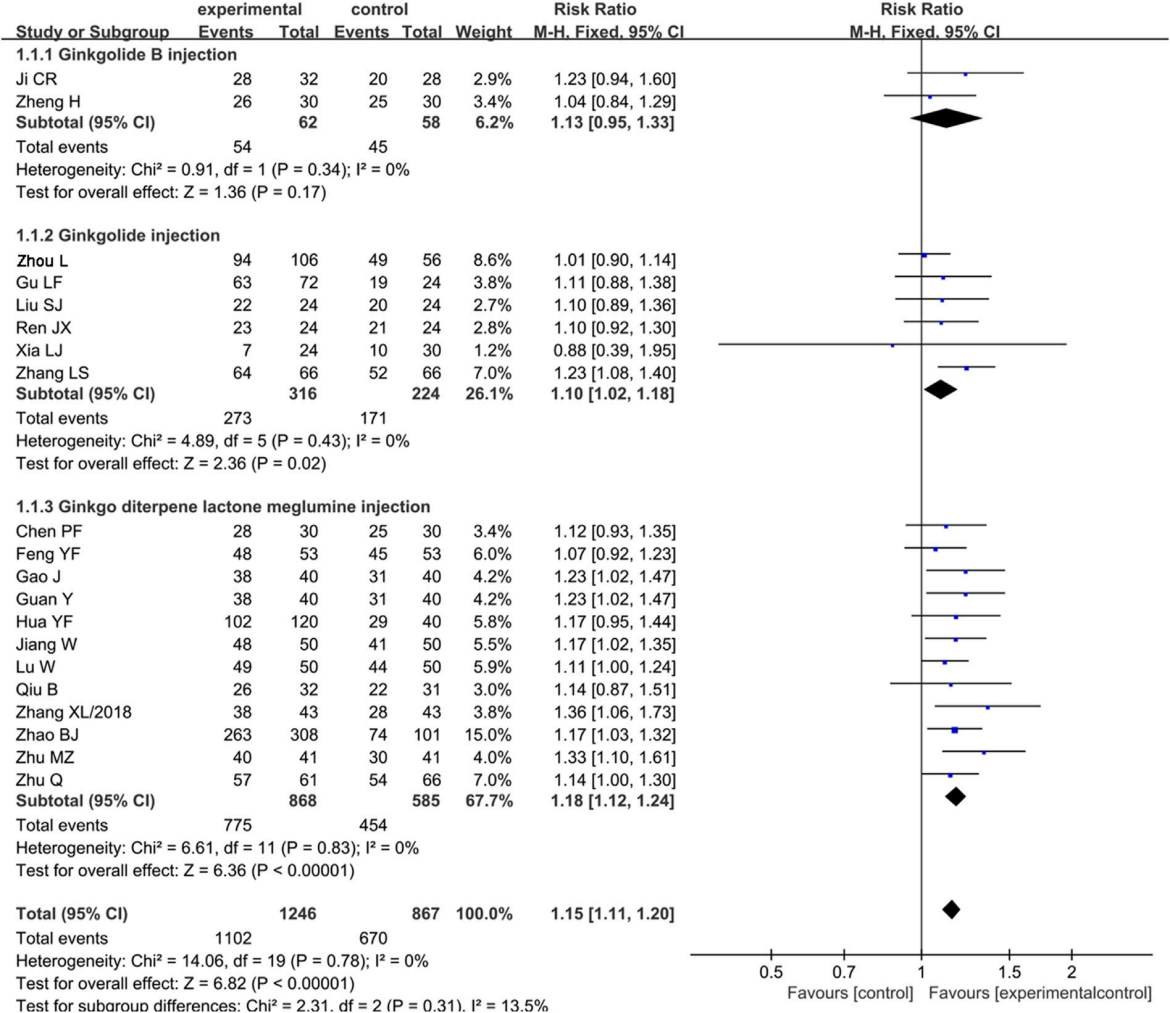
Forest plot of ginkgo terpene lactone preparations on clinical efficacy.

A total of 20 articles were identified which compared the clinical efficacy of ginkgo terpene lactone injections with that of other drugs for further subgroup analysis. In brief, two articles reported the efficacy of ginkgolide B injection with no statistical heterogeneity (*p* = 0.34, I^2^ = 0%), so the fixed effect model was employed for analysis. The results showed that clinical efficacy of ginkgolide B injection in the treatment of IS was similar to that of the control group [RR = 1.13, 95% CI (0.95, 1.33), Z = 1.36, *p* = 0.17]. A total of six trials reported the clinical efficacy of ginkgolide injection in treatment of IS, following the test of heterogeneity (*p* = 0.43, I^2^ = 0%), so the fixed effect model was employed for analysis. The results showed that ginkgolide injection achieved better clinical efficacy [RR = 1.10, 95% CI (1.02, 1.18), Z = 2.36, *p* = 0.02]. Moreover, the heterogeneity of results was not statistical in 12 trials which reported the effectiveness of ginkgo diterpene lactone meglumine injection (*p* = 0.83, I^2^ = 0%), so the fixed effect model was adopted. The results showed that the clinical efficacy of ginkgo diterpene lactone meglumine injection was superior to that of control group [RR = 1.18, 95% CI (1.12, 1.24), Z = 6.36, *p* < 0.001] (Figure 3).

#### Changes of Neurological Function Deficit

**FIGURE 4:**
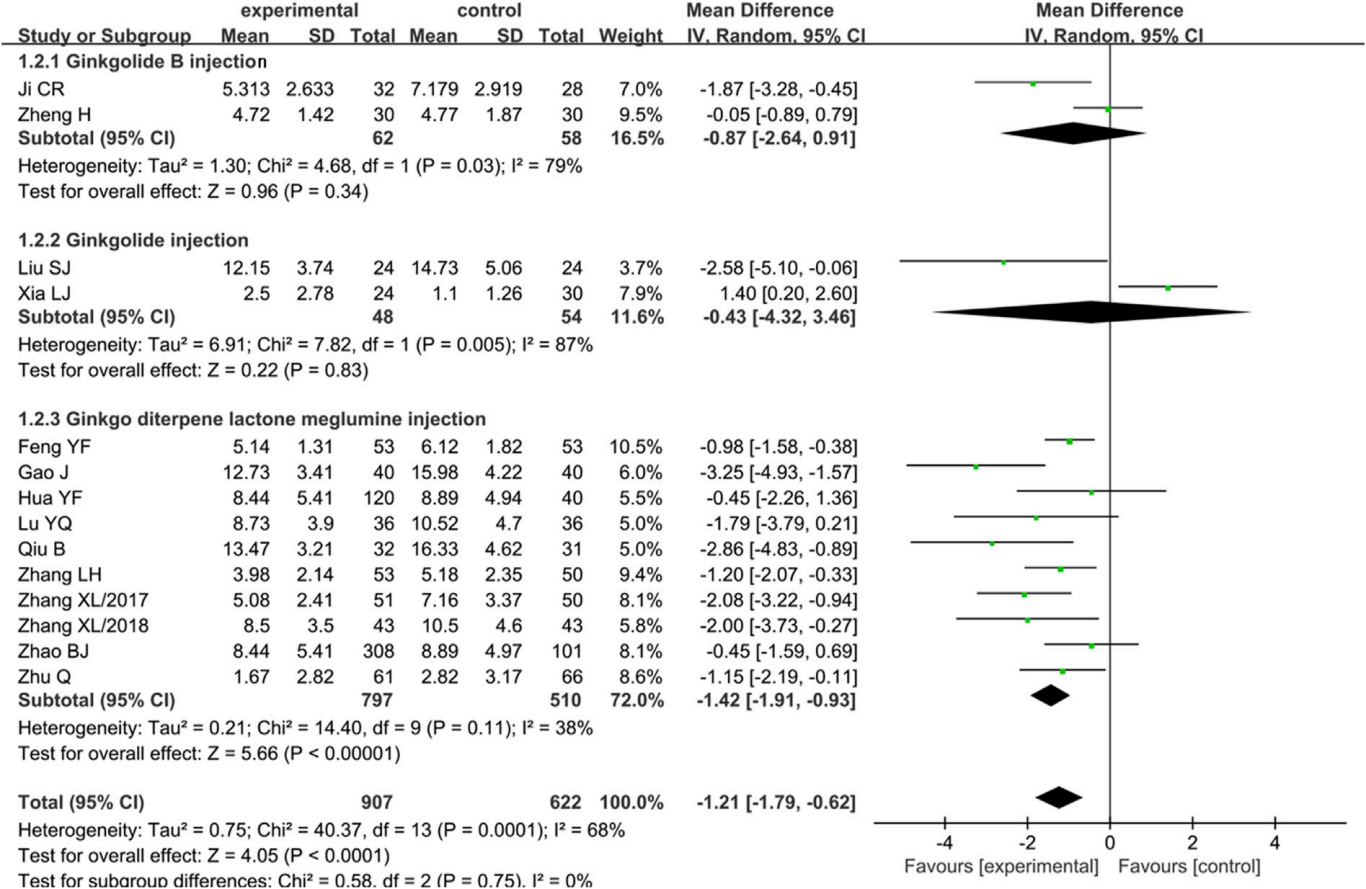
Forest plot of ginkgo terpene lactone preparations on the changes of neurological function deficit.

In total, 14 articles assessed the effects of ginkgo terpene lactone preparations on changes of neurological function deficit by NIHSS or NFDS. Among the articles, the heterogeneity of results was statistical (*p* = 0.0001, I^2^ = 68%), so the random effect model was adopted. To be specific, two articles reported the changes of neurological function deficit of ginkgolide B injection, and the results showed that the effect of ginkgolide B in improving neurological function was similar to that of the control group [MD = -0.87, 95% CI (-2.64, 0.91), Z = 0.96, *p* = 0.34]. Additionally, two trials reported the changes of neurological function deficit of ginkgolide injection and found that the effect of ginkgolide injection in improving neurological function was similar to that of the control group [MD = −0.43, 95% CI (−4.32, 3.46), Z = 0.22, *p* = 0.83]. Moreover, 10 trials reported the changes of neurological function deficit of ginkgo diterpene lactone meglumine injection and found that ginkgo diterpene lactone meglumine injection achieved better effects in improving neurological function [MD = −1.42, 95% CI (−1.91, −0.93), Z = 5.66, *p* < 0.001] (Figure 4).

#### Analysis of Safety

**FIGURE 5:**
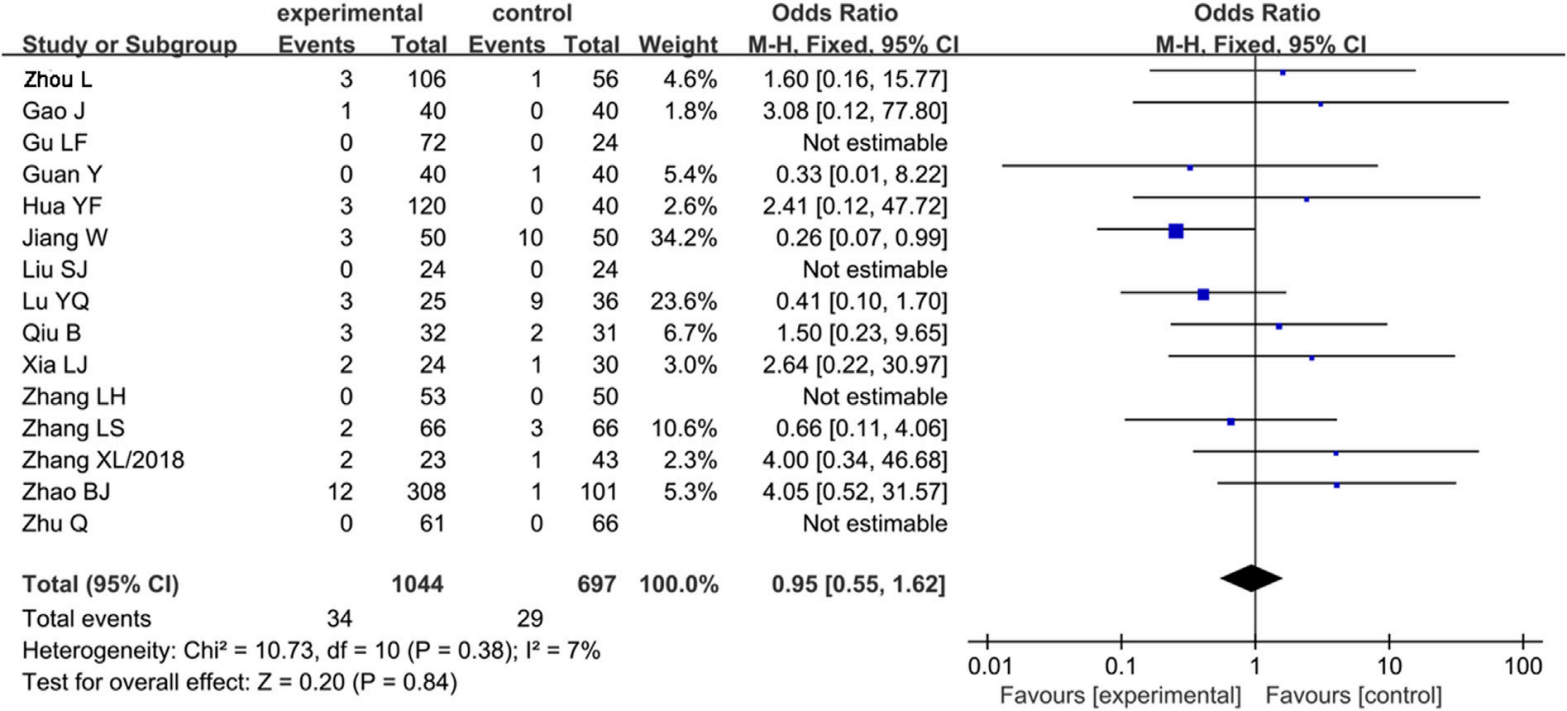
Forest plot of ginkgo terpene lactone preparations on ADRs/ADEs; ADRs/ADEs, adverse drug reactions/adverse drug events.

Among the 23 included references, there were 15 references which reported the ADRs/ADEs including skin rash, nausea, fatigue, chest tightness, and palpitations. However, all ADRs/ADEs were disappeared when the treatment was over. There were no obvious changes in the liver and kidney function before and after treatment. The rate of ADRs/ADEs was analyzed, and the results showed that it was similar between the experiment group and control group [OR = 0.95, 95% CI (0.55, 1.62), Z = 0.20, *p* = 0.84] (Figure 5).

#### Analysis of Risk of Publication Bias

To evaluate the risk bias of 20 articles on the clinical efficacy and 14 articles on the changes of neurological function deficit, funnel plots were used. As shown in Supplemental Figures S1, S2, most of the data were concentrated at the top, and the images showed basic symmetry. Furthermore, the absence of the publication bias was supported by Egger’s test (*p* = 0.21, *p* = 0.32).

### Meta-Analysis of Ginkgo Diterpene Lactone Meglumine Injection Combined With Thrombolytic Therapy in Treatment for IS

A total of five articles involving ginkgo diterpene lactone meglumine injection combined with rt-PA were included to explore its add-on effects on treatment for acute IS.

#### Analysis of Clinical Efficacy

**FIGURE 6:**
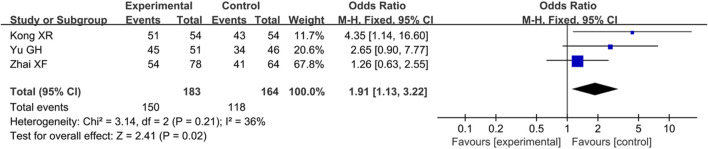
Forest plot of add-on effects of ginkgo diterpene lactone meglumine injection on clinical efficacy.

In total, three articles reported the clinical efficacy with no statistical heterogeneity (*p* = 0.21, I^2^ = 36%), so the fixed effect model was employed for analysis. The results showed that the clinical efficacy of ginkgolide diterpene lactone meglumine injection combined with rt-PA was better than that of rt-PA alone [OR = 1.91, 95% CI (1.13, 3.22), Z = 2.41, *p* = 0.02] (Figure 6).

#### Changes of Neurological Function Deficit

**FIGURE 7:**
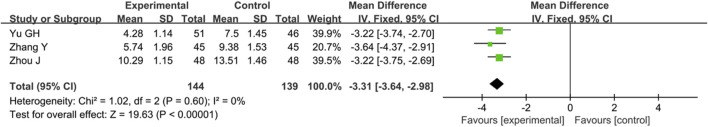
Forest plot of add-on effects of ginkgo diterpene lactone meglumine injection on changes of neurological function deficit.

In total, three articles reported the changes of neurological function deficit with no statistical heterogeneity (*p* = 0.60, I^2^ = 0%), so the fixed effect model was utilized. The results showed that ginkgo diterpene lactone meglumine injection combined with rt-PA in improving neurological function was superior to only rt-PA [MD = −3.31, 95% CI (−3.64, −2.98), Z = 19.63, *p* < 0.001] (Figure 7).

#### Analysis of Safety

**FIGURE 8:**
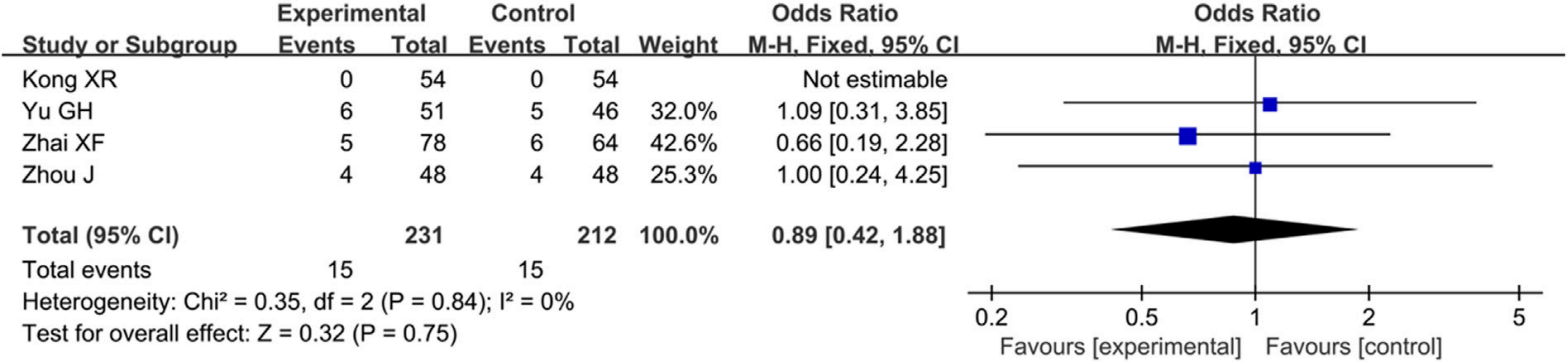
Forest plot of meta-analysis of add-on effects of ginkgo diterpene lactone meglumine injection on ADRs/ADEs; ADRs/ADEs, adverse drug reactions/adverse drug events.

Among the five included articles, there were four articles that reported the ADRs/ADEs including nausea, fatigue, chest tightness, palpitations, and hemorrhage transformation. As presented in Figure 8, the rate of ADRs/ADEs was similar between the experiment group and control group [OR = 0.89, 95% CI (0.42, 1.88), Z = 0.32, *p* = 0.75].

## Discussion

In this systematic review, we found that ginkgo terpene lactone injections have good therapeutic effects on patients with IS, especially ginkgo diterpene lactone meglumine injection was more effective for IS than other types of drugs in the aspects of improving clinical efficacy and neurological function. Furthermore, the combination of ginkgo diterpene lactone meglumine injection and rt-PA was superior to only rt-PA for clinical efficacy, supporting add-on effects of ginkgo terpene lactone preparations in thrombolytic therapy on acute IS.

Ginkgo terpene lactones, the main active ingredients from *Ginkgo* leaves (Geng et al., 2018), have the effects of blocking PAF activity (Liu et al., 2018), anti-inflammatory and antioxidant activities (Gong et al., 2014), and regulating excitatory amino acids and brain cell energy metabolism (Yang et al., 2011). Initially, *Ginkgo biloba* extract (EGb761) has been originated by Dr Willmar Schwabe Pharmaceuticals which contains approximately 24% flavone glycosides and 6% terpene lactones (ginkgolides A, B, and C and bilobalide) (Nash and Shah, 2015). Ginaton and Shuxuening injections are the typical representatives of EGb761, which are still widely used in the treatment of IS. However, EGb761 contains a low content of terpene lactones and multiple medicinal components with complex interactions (DeFeudis, 1998). With the development of pharmaceutical technology, high-purity injections have been proposed to reduce the incidence of adverse reactions. Ginkgo diterpene lactone meglumine injection is one of the monomer injections that have been approved for marketing, which includes effective ingredients of ginkgolides A, B, and K with advantages of high content of active ingredients and low adverse reactions (Zhou et al., 2017). Additionally, previous studies have demonstrated that ginkgolide injection and ginkgolide B can protect against IS as well (Nabavi et al., 2015; Xiang et al., 2021). In this meta-analysis, we further found that clinical efficacy of ginkgo terpene lactone injections (especially ginkgo diterpene lactone meglumine injection) was superior to that of other drugs in the treatment of IS including ginkgo leaf extract and dipyridamole injection and Shuxuening injection. Additionally, none of the included studies reported serious ADRs/ADEs. Thus, ginkgo terpene lactone injections, especially ginkgo diterpene lactone meglumine injection may be deemed as better choices for treating IS in clinical practice.

For management of patient with acute IS, intravenous rt-PA was the approved therapy within 4.5 h from the symptom onset (Prabhakaran et al., 2015). However, rt-PA thrombolysis can lead to not only destroying the blood–brain barrier but also producing cytotoxic brain edema (Yepes et al., 2009). Therefore, how to improve the adverse effects and clinical efficacy is also a major point of this study. Previously, a meta-analysis has demonstrated that *Ginkgo biloba* leaf preparation combined with aspirin can help to avoid adverse reactions from high-dose aspirin and concluded that *Ginkgo biloba* leaf preparation can be used as a complementary therapy (Zhao et al., 2021). Based on add-on effects, we analyzed the five trials of ginkgo diterpene lactone meglumine injection combined with rt-PA. The results showed that combination therapy was superior to rt-PA alone in improvement of clinical efficacy. Correspondingly, the previous study (Hu, 2018) has stated that *Ginkgo biloba* extract can alleviate the neurotransmitter metabolism and energy and amino acid disturbances induced by rt-PA. Therefore, while rt-PA is the preferred drug for the first prevention of acute IS, ginkgo diterpene lactone meglumine injection can be used as a complementary therapy.

There were some limitations in this study. In brief, although this systematic review carried out a comprehensive search, it cannot be ruled out that some of the gray literatures were not included. Additionally, most of the included trials were RCTs in a single center with small samples, so there was the possibility of bias. In future, a multicenter, large sample size, and double-blind placebo-controlled design is needed to verify the reliability of this meta-analysis.

## Conclusion

Ginkgo terpene lactone preparations have good therapeutic effects on patients with IS. For acute IS, ginkgo diterpene lactone meglumine injection can be used as a complementary therapy to improve the clinical efficacy of rt-PA. However, further large and rigorous trials should be also warranted.

## Data Availability

The original contributions presented in the study are included in the article/Supplementary Material, further inquiries can be directed to the corresponding author.
