# Supplementary figures and images for: The Efficacy and Safety of Ginkgo Terpene Lactone Preparations in the Treatment of Ischemic Stroke: A Systematic Review and Meta-Analysis of Randomized Clinical Trials
Source: Front Pharmacol. 2022 Mar 18;13:821937. doi: 10.3389/fphar.2022.821937 (PMC8982077; doi:10.3389/fphar.2022.821937)

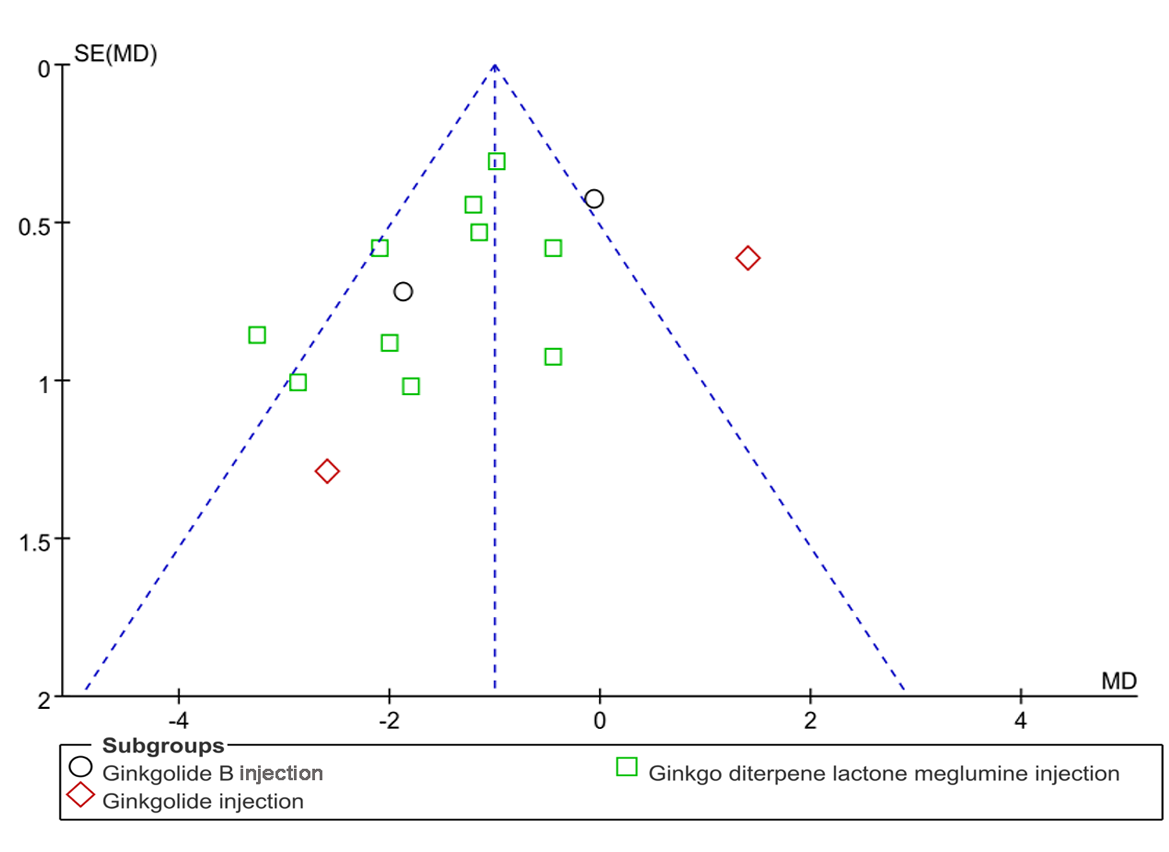

Supplement: Supplementary file 1 [file Image2.TIF]

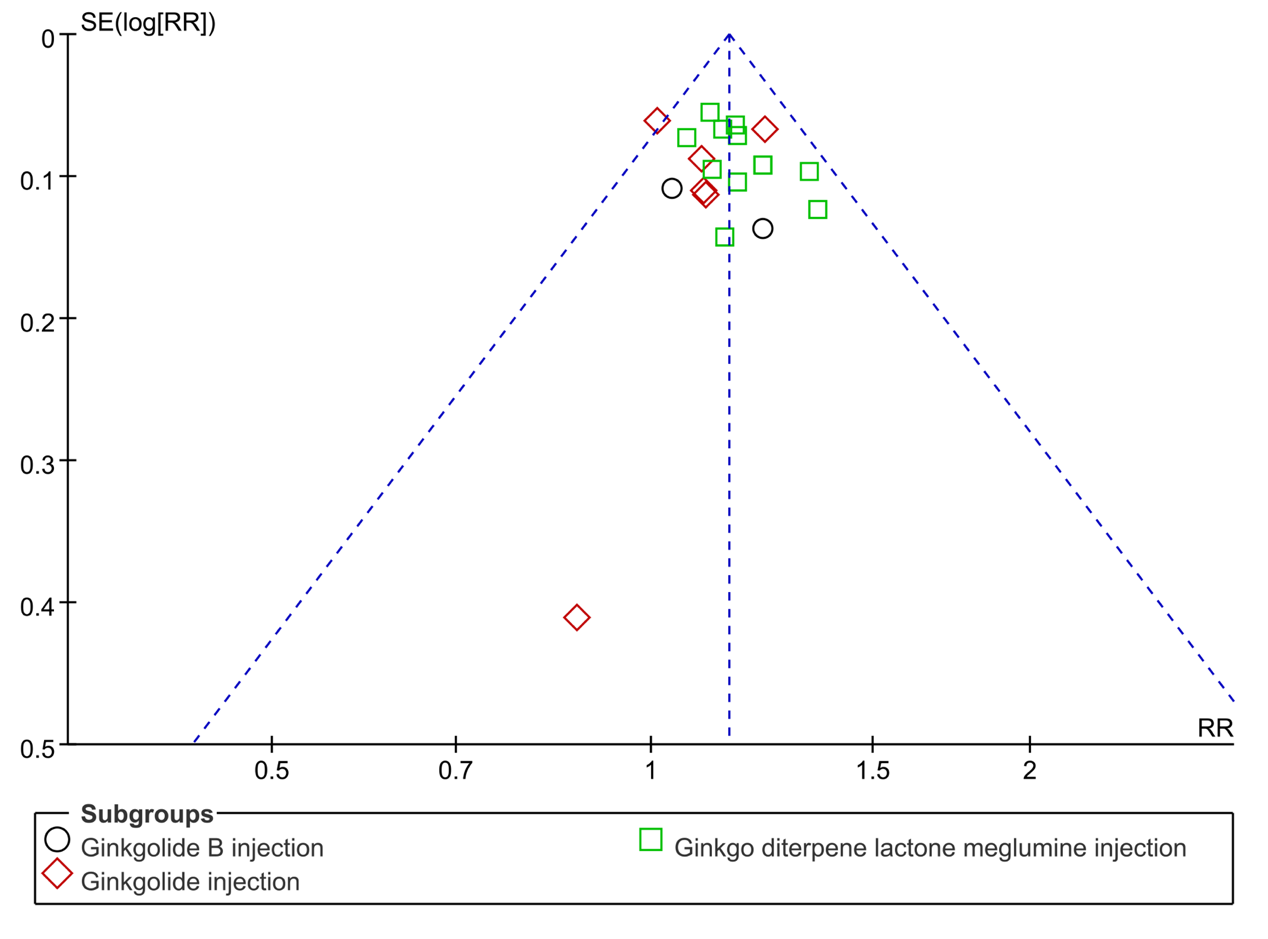

Supplement: Supplementary file 2 [file Image1.TIF]
